# Ensembl 2016

**DOI:** 10.1093/nar/gkv1157

**Published:** 2015-12-19

**Authors:** Andrew Yates, Wasiu Akanni, M. Ridwan Amode, Daniel Barrell, Konstantinos Billis, Denise Carvalho-Silva, Carla Cummins, Peter Clapham, Stephen Fitzgerald, Laurent Gil, Carlos García Girón, Leo Gordon, Thibaut Hourlier, Sarah E. Hunt, Sophie H. Janacek, Nathan Johnson, Thomas Juettemann, Stephen Keenan, Ilias Lavidas, Fergal J. Martin, Thomas Maurel, William McLaren, Daniel N. Murphy, Rishi Nag, Michael Nuhn, Anne Parker, Mateus Patricio, Miguel Pignatelli, Matthew Rahtz, Harpreet Singh Riat, Daniel Sheppard, Kieron Taylor, Anja Thormann, Alessandro Vullo, Steven P. Wilder, Amonida Zadissa, Ewan Birney, Jennifer Harrow, Matthieu Muffato, Emily Perry, Magali Ruffier, Giulietta Spudich, Stephen J. Trevanion, Fiona Cunningham, Bronwen L. Aken, Daniel R. Zerbino, Paul Flicek

**Affiliations:** 1 European Molecular Biology Laboratory, European Bioinformatics Institute, Wellcome Genome Campus, Hinxton, Cambridge CB10 1SD, UK; 2 Wellcome Trust Sanger Institute, Wellcome Genome Campus, Hinxton, Cambridge, CB10 1SA, UK

## Abstract

The Ensembl project (http://www.ensembl.org) is a system for genome annotation, analysis, storage and dissemination designed to facilitate the access of genomic annotation from chordates and key model organisms. It provides access to data from 87 species across our main and early access Pre! websites. This year we introduced three newly annotated species and released numerous updates across our supported species with a concentration on data for the latest genome assemblies of human, mouse, zebrafish and rat. We also provided two data updates for the previous human assembly, GRCh37, through a dedicated website (http://grch37.ensembl.org). Our tools, in particular the VEP, have been improved significantly through integration of additional third party data. REST is now capable of larger-scale analysis and our regulatory data BioMart can deliver faster results. The website is now capable of displaying long-range interactions such as those found in *cis*-regulated datasets. Finally we have launched a website optimized for mobile devices providing views of genes, variants and phenotypes. Our data is made available without restriction and all code is available from our GitHub organization site (http://github.com/Ensembl) under an Apache 2.0 license.

## INTRODUCTION

Ensembl (http://www.ensembl.org) generates genomic datasets through a system that is designed to analyse, store and distribute data, and which enables interpretation through open data release. While acting as a hub of reference and baseline data similar to the UCSC Genome Browser (1) and RefSeq (2), we also distribute datasets we create and promote standards and interoperability between genomic resources. We engage with the scientific community through an active outreach program and helpdesk. In addition we collaborate with and often play active leadership roles in projects such as ENCODE (3), the Genome Reference Consortium (GRC) (4), the Global Alliance for Genomics and Health (GA4GH) and GENCODE (5). Ensembl is updated four to five times per year with each release representing a data and software freeze. This procedure ensures that all our data are consistent, no matter the method of access. Every release is accompanied by archived versions of our website and BioMart data mining tool with a three year rolling retention policy. All public data releases regardless of age are available from our FTP site, MySQL servers and public Git repositories. In addition a REST API provides program language agnostic access to the current data release.

Our analysis methods construct annotation through the processing and summarization of experimental evidence. Gene annotation relies on the alignment of cDNAs and proteins from resources such as RefSeq and UniProt (6) alongside building transcription models from RNA-seq alignment data. Our Regulatory Build is based on high quality experimental evidence from projects such as ENCODE and Roadmap Epigenomics (7) and is capable of annotating a diverse set of features across many distinct cell types (8). All gene and regulatory annotation is accessioned and versioned between releases enabling downstream analysis to accurately refer back to these annotations. We also produce comparative genomics resources, which build on top of this gene annotation to calculate gene evolution and orthology information and use genomic DNA to build whole genome pairwise and multiple sequence alignments. Finally our variation resources integrate disparate data sources (including dbSNP (9), HGMD (10), ClinVar (11)) and present them through a consistent integrated interface. Variant consequences are calculated with reference to our gene and regulatory annotation and quantified by standard protein consequence analyses.

Annotation is made available through a set of mature Perl Application Programming Interfaces (APIs), which broker data from our databases. These same APIs are used to build our website and analysis methods and they are available externally for others to use in building their own methods and tools. Our infrastructure, whilst originally developed to operate on chordates and core model organisms, has been successfully deployed for a wide range of taxa as shown by our sister project Ensembl Genomes (12). Tools such as the Ensembl Variant Effect Predictor (VEP) can be applied to any genome due to our common programming interface and support for standard data formats (13). All Ensembl software is available under an Apache 2.0 license and is free for all to use.

Release 82 (September 2015) makes 69 species available from our main website, 18 species from our pre-release website (http://pre.ensembl.org) and GRCh37 annotation served from a dedicated website (http://grch37.ensembl.org). All chordates have been analysed by our gene annotation methods. Variation data is available for 22 species and regulatory data is available for human and mouse. All three websites provide sequence search. Additional tools are available on our main and GRCh37 websites, including the VEP and an assembly coordinate conversion tool based on CrossMap (14). The main website is hosted from four locations based in the UK, Singapore, US East and West coasts; the final three are deployed on Amazon Web Services. We also provide the ability to attach standard bioinformatic data formats including BigBED, BigWig (15), VCF (16) and BAM (17) to visualize external data in the context of our own data and support the UCSC track hub format to orchestrate track configuration (18).

This report focuses on new data and important technological changes to the project. We explain how these improvements enhance Ensembl and aid the analysis and interpretation of genomic data.

## GENOME ANNOTATION

### Protein coding and non-protein coding gene annotation

Over the past year, we have concentrated on supporting our most accessed species and annotating a selection of new genomes. As a member of the GENCODE consortium, we have followed its recent decision to improve mouse gene annotation to a similar level to human and have adopted a new gene annotation release cycle. Computationally annotated mouse gene annotation is currently merged every release with manual annotation from the HAVANA project (19). The human genome receives an update every other release with zebrafish or rat receiving gene annotation updates in those releases when human does not. We have incorporated several minor assembly updates (three for human and two for mouse) including GRCh38.p3 and GRCm38.p4. Both were released in Ensembl 81 (July 2015). The human and mouse gene annotations are supplemented by three methods to help quantify transcript support and provide subsets of the GENCODE dataset. Transcript support levels (TSL) are an expression of how well mRNA and EST libraries align to transcripts across splice junctions. Transcripts are assigned a numeric value from one to five indicating the level of support. APPRIS is used to identify principle transcript isoforms of genes from proteomic datasets (20). Finally the GENCODE Basic representative transcript set prioritizes full-length protein coding transcripts over partial or non-protein coding transcripts and is based on rules agreed by the GENCODE consortium (21).

In addition, we have annotated two major assembly updates for rat (Rnor_6.0) and zebrafish (GRCz10). Both gene sets include manual annotation from the HAVANA project. We have also recently updated our lincRNA annotation methods to using candidate transcript models built from RNA-seq data. These are tested for protein coding potential by searching for Pfam domains and alignments to the UniProt database. Models that show no protein coding potential are labelled as lincRNA. We have applied this method to generate lincRNAs for rat and sheep and aim to extend the method to other species over the coming year. We have also produced preliminary transcript models for Crab-eating macaque (*Macaca fascicularis*) and sperm whale (*Physeter macrocephalus*) by aligning experimental data and homologous proteins; these are available on our Pre! website.

To compare our annotation to external gene sets we have imported both Consensus CDS (CCDS) and RefSeq transcripts for human, mouse and selected other species, into our infrastructure (22). We annotate RefSeq transcripts when their mRNA sequence does not exactly match the underlying genomic sequence and provide details on where the mismatch or insertion-deletion occurs e.g. 5′ UTR, CDS, 3′ UTR. RefSeq transcripts that exactly match an overlapping Ensembl-generated model, with respect to the entire model or just coding exons, are annotated accordingly. All annotation is available to downstream analysis tools including the VEP and via our unified APIs.

### Variation annotation

Our variation resources integrate essentially all publicly available germline and somatic variant data for 20 vertebrate species. Over the past year the number of SNPs, indels and structural variants in the databases has almost doubled to 468 million variants. We have seen a dramatic increase in genotypes for human (206 000 million), cow (160 million) and sheep (6300 million). This led us to develop a new VCF-based genotype layer to reduce storage, processing and access time for these data. In addition, we have redesigned the variation database schema to model individuals with multiple samples.

Alongside variation data we also bring in phenotype, trait and disease annotations for 14 species totalling 2.8 million annotations of genes, short variants, structural variants and QTLs. For human these data span over 15 000 phenotypes, traits and diseases from 17 sources including ClinVar, OMIM (Online Mendelian Inheritance in Man) (23) and the NHGRI-EBI GWAS Catalog (24). Eight sources are incorporated for other species including RGD (25), OMIA (26), AnimalQTL (27), ZFIN (28).

In addition to the above work we supplement our data with available citations, pass variants though our quality-control procedures and predict the consequence of variants on our gene sets and regulatory regions. For every possible amino acid change in our 10 most popular species, we run SIFT with enhanced quality information (29) and PolyPhen-2 (30) (human only). We also compute Human Genome Variation Society (HGVS) nomenclature for every variant and recently moved to 3′ shifting of indels to conform to the HGVS specification.

### Regulatory annotation

Ensembl Regulation annotation describes the functional role of non-genic genomic elements, in particular enhancers, promoters and insulators, using biochemical assays such as ChIP-Seq or DNAse1 hypersensitivity. The re-designed method, which was deployed last year on 18 human cell lines, has been extended to mouse and covers 8 cell lines and tissues (8).

**Figure 1.**
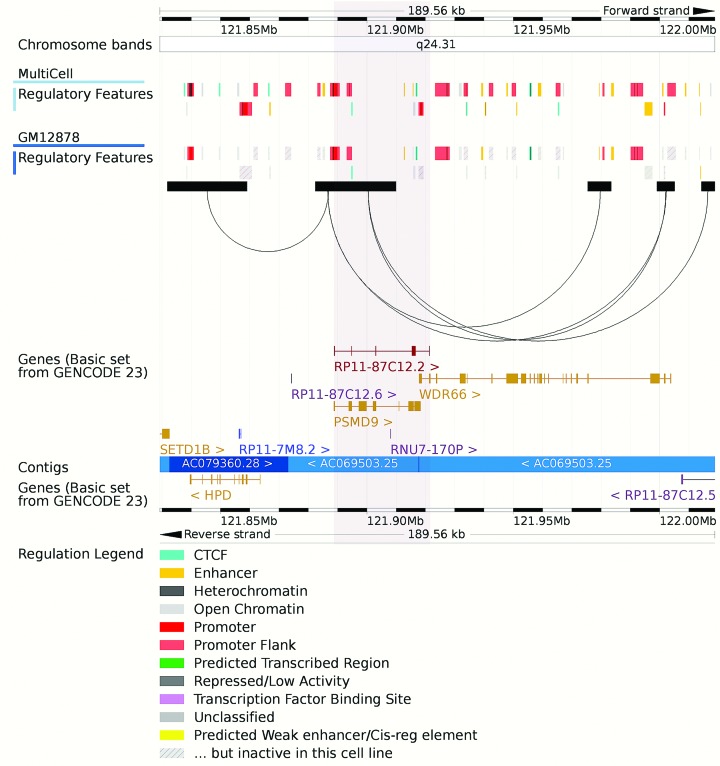
Ensembl's location view showing the drawing of long-range interaction arcs and new region marking tool. The grey boxes indicate HindIII fragments and the arcs represent selected significant interactions between promoters and their distal interacting elements, measured using high resolution Capture Hi-C in the GM12878 lymphoblastoid cell line and displayed for the GRCh38 assembly (43). The summary Ensembl Regulatory Build and the GM12878 specific regulatory activities are also shown. Our region marking tool is shown as a light grey box surrounding the transcripts PSMD9 and RP11–87C12.2.

In anticipation of high-level *cis*-regulatory datasets, which will link regulatory elements to neighbouring genes, we can now render interaction data on our graphical genome location view. Interaction elements are described by the existing WashU Epigenome Browser formats and then loaded onto our website via user upload or by specifying a HTTP URL (31). The data are then visualized as arcs spanning across the region in question, as illustrated in Figure 1.

### Comparative annotation

Ensembl's comparative analysis integrates the genome sequences and gene annotations of all available species into a single comprehensive resource. We have updated our whole-genome alignments due to updates to both rat and zebrafish assemblies. The zebrafish assembly update resulted in re-computing 20 pairwise whole genome alignments and our fish Enredo Pecan Ortheus (EPO) multiple alignments (32). We have also retired our fish-specific EPO method resulting in a single EPO production pipeline applicable to fish, mammal and sauropsid multiple sequence alignments.

Major development work is on going to move towards a new protein clustering and classification system. Our current method is based on clustering blastp distances using hcluster_sg (33). It will be replaced with a more straightforward HMM classification based upon PANTHER (34). Moving to an HMM classification will enable analysis and clustering of proteins in linear time compared to our previous approach. We are also developing two methods of gene tree reconstruction. The first constructs gene trees *de novo* whilst the second enables gene tree modification by removing genes or inserting new genes into the tree. Our new methods are being benchmarked via the Quest for Orthologues service (http://orthology.benchmarkservice.org), which tests a range of metrics including tree-consistency approaches, gene ontology and enzyme classification tests (35). These developments will address the increasing numbers of species available for comparative analysis in Ensembl and Ensembl Genomes and improve the stability of the predicted gene-trees and orthologies.

### GRCh37 human assembly support

Our GRCh37 website, supporting the previous human assembly, has received two major releases over the past year. Our first (March 2015) included new data variant from the 1000 Genomes Project phase 3 (36), dbSNP, COSMIC v71 (37) and HGMD. The second update (October 2015) incorporated variants from the Exome Aggregation Consortium (ExAC) (38) and NHLBI Exome Sequencing Project (39). A full selection of resources is available for GRCh37 including BioMart and public MySQL access. Our FTP site (ftp://ftp.ensembl.org/pub/grch37) provides VEP cache and FASTA updates. We also maintain a GRCh37 REST API hosted at http://grch37.rest.ensembl.org.

## WEBSITE, TOOLS AND INFRASTRUCTURE

### VEP

This year we have improved the VEP's ability to report transcript attributes such as a transcript's existence in the GENCODE Basic set and a transcript's TSL support level (both subject to availability). We also record, in the VEP's output, if phenotype/disease data are available. Additionally, it is possible to report predictions on RefSeq transcripts and the VEP will now indicate if the transcript matches a model annotated by Ensembl. Finally the VEP now supports selenocysteine modifications.

The standalone VEP has been enhanced by several new plugins. It is now possible to retrieve ExAC allele frequencies from downloaded VCFs, to query for splice site predictions from dbscSNV (40) and finally to retrieve gene expression levels from Gene Expression Atlas data via their web service API (41). Other plugins allow the VEP to locate the nearest gene to a variant and indicate if a variant has been shifted in HGVS notation. Our online tool interface has also been updated to provide an immediate overview of a single variant's consequences. The ‘Instant VEP’ tool queries our live REST API to return consequence data in less than a second.

Extensive development work on the VEP has resulted in significant reductions in runtime. For example, analysis of NA12878 from Illumina's Platinum Genome dataset (annotating 4 498 138 variants) using the GRCh38 assembly and release 81 data took 113 min to complete using four compute cores (42). The same analysis performed using release 77 data and analysis, October 2014, took 199 min to complete. To improve the installation procedure we now quality control all downloads using an automatic checksum computation. We have also improved the installation script's warning handling. In addition version information has been added to the cache to improve debugging and data source tracking.

### Web

This year has seen a number of incremental improvements to our website. Data export has been re-engineered to simplify extracting data from our resource and now enables the download of sequences, pairwise alignments, multiple alignments, orthologues and gene-trees. We also provide improved high-resolution images for publication and high contrast images for use in presentations. User data import has been enhanced through better support for UCSC Track Hubs and we now accept hubs with data from multiple species. Additionally we support composite tracks and have improved track labelling. Finally our website supports track visibility settings allowing a hub to have a number of tracks enabled by default.

**Figure 2.**
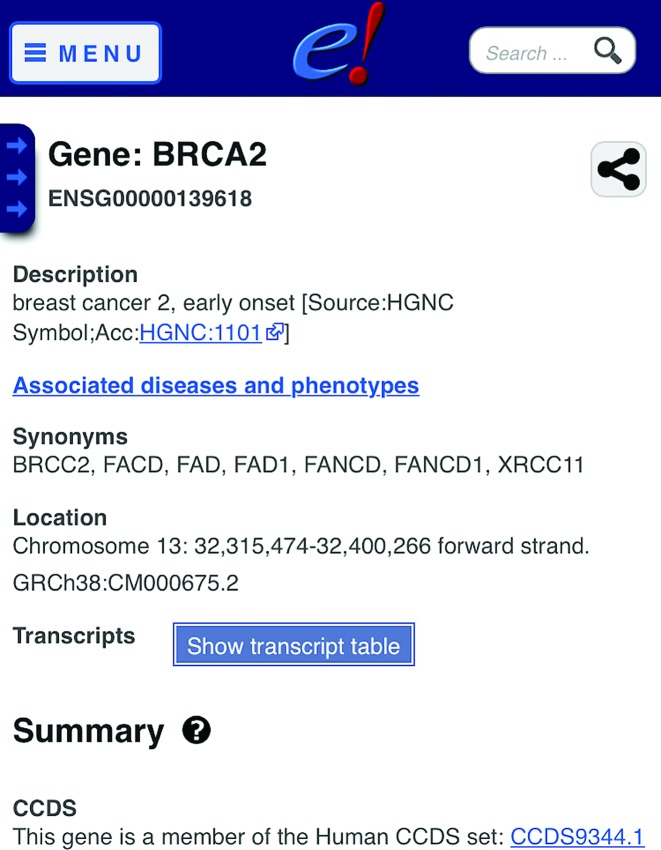
The new Ensembl mobile site showing BRCA2 and detailing available synonyms, genomic location and links to external resources such as CCDS. Search is available in the top right corner on all mobile site pages and each page has the ability to be shared over social media and email.

Gene Expression Atlas (GXA) baseline expression data is integrated into our website using a GXA provided JavaScript widget. Gene expression baseline levels are available for a number of studies including FANTOM5 (44) and GTEx (45). Our location view has been enhanced with two new interaction modes. In ‘Select’ mode, clicking and dragging with the mouse will select a resizable region and present a menu with options to either zoom or mark a region. The marked area is shown as a grey box and will remain marked until actively removed. This mark persists into our image exports as demonstrated in Figure 1. In ‘Drag’ mode, clicking on the image enables rudimentary scrolling navigation dragging the image to the left or right. Finally we released a new mobile optimized website, as shown in Figure 2, which can be accessed at http://m.ensembl.org or via optional redirection from our main website. The website is optimized for reduced display sizes and offers targeted views of genes, variants and phenotypes. Mobile users can opt to return to the full site when they require more advanced functionality.

### REST service

The last year has seen substantial growth in both data and usage for our programming language agnostic REST API (46). All DNA from the previous five human assemblies (versions NCBI34 to GRCh38) are available by specifying the desired assembly version. Omitting a version assumes the latest assembly. Our VEP endpoint now supports annotation using HGVS variant nomenclature (e.g. AGT:c.803T>C) and querying for variants from a protein has been significantly improved. Building on our release in 2014, eight endpoints now support batch querying via the HTTP POST method including our sequence, identifier lookup and archive endpoints. We also support a number of GA4GH methods for retrieving sample genotype calls, variant calls on a reference sequence and for discovering available variant datasets and are actively working on a GA4GH variant annotation prototype.

### eHive

eHive is the pipeline management system that powers a significant proportion of our compute, over 300 CPU years of compute per year and is versioned outside of the Ensembl release cycle (47). This year we have released version 2.3, which now supports a generic guest language interface to facilitate the writing of runnables in languages other than Perl through a standardized interprocess communication protocol. We have written a reference implementation in Python allowing Python and Perl code to be executed in the same workflow. Support for Java is under active development. Finally version 2.3 enhances our standard modules by improving their ability to capture and respond to erroneous system commands alongside improved security when interacting with databases. All development is continuously tested on Travis CI (a public continuous integration service) with 70% of the code tree covered by tests.

### BioMart

Our BioMart databases continue to be updated every release in order to provide the latest annotation and imported data (48). We have made protein domain coordinates, transcript length and the previously described GENCODE Basic, TSL and APPRIS datasets available. Our relationship with biomaRt and Bioconductor/R has resulted in the release of dedicated subroutines to allow R developers to easily query our live, GRCh37 and archive BioMart services (49). Finally, in release 79 (March 2015), we redesigned our regulation BioMart to improve query performance and to meet the projected demands in data volumes. We have created seven datasets targeting distinct classes of data such as regulatory features as annotated by our methods, as well as binding motifs and miRNA targets. Consequently querying for data restricted by genomic location can be retrieved six times faster than using our previous BioMart.

## OUTREACH AND TRAINING

External user support is provided by means of face-to-face training courses, online training materials, social media and email help channels. Annually we deliver roughly 100 workshops at research institutes and conferences around the world in person and through live webinars. Online training covering five Ensembl courses is available through the EMBL-EBI Train Online interface (http://www.ebi.ac.uk/training/online/subjects/11), while our YouTube channel contains 35 training videos (https://www.youtube.com/user/EnsemblHelpdesk). Training material is also available via our help pages and workshops can be requested via our helpdesk.

Queries about working with Ensembl data, interfaces and APIs can be directed to our helpdesk (helpdesk@ensembl.org) or our public developers mailing list (dev@ensembl.org). We are active on social media channels such as Twitter (https://twitter.com/ensembl), Facebook (https://www.facebook.com/Ensembl.org) and our blog (http://www.ensembl.info/). For example, we regularly use #citedEnsembl hashtag on Twitter to highlight published research that has used Ensembl resources.
